# Nomogram using human epididymis protein 4 predicted concurrent endometrial cancer from endometrial atypical hyperplasia before surgery

**DOI:** 10.3389/fonc.2024.1442127

**Published:** 2024-09-06

**Authors:** Yaochen Lou, Feng Jiang, Yan Du, Jun Guan

**Affiliations:** ^1^ Department of Gynecology, Obstetrics and Gynecology Hospital of Fudan University, Shanghai, China; ^2^ Department of Neonatology, Obstetrics and Gynecology Hospital of Fudan University, Shanghai, China; ^3^ Clinical Research Unit, Obstetrics and Gynecology Hospital of Fudan University, Shanghai, China

**Keywords:** human epididymis protein 4, menopausal status, endometrial cancer, endometrial atypical hyperplasia, predictive factor

## Abstract

**Objective:**

To establish a nomogram based on presurgical predictors of concurrent endometrial cancer (EC) for patients diagnosed with endometrial atypical hyperplasia before definitive surgery (preoperative-EAH) to improve the risk stratification and clinical application.

**Methods:**

Preoperative-EAH patients who underwent hysterectomy in a tertiary hospital from January 2020 to December 2022 were retrospectively analyzed. Independent predictors from the multivariate logistic regression model were used to establish a nomogram, and bootstrap resampling was used for internal validation.

**Results:**

Of 370 preoperative-EAH patients, 23.4% were diagnosed with EC after definitive surgery (final-EC). Multivariate analyses found three independent predictors of final EC: human epididymis protein 4 (HE4) ≥43.50 pmol/L [odds ratio (OR) = 3.70; 95% confidence intervals (CI) = 2.06–6.67], body mass index (BMI) ≥ 28 kg/m^2^ (OR = 2.05; 95% CI = 1.14–3.69), and postmenopausal status, particularly at postmenopausal time ≥5 years (OR = 5.84, 95% CI = 2.51–13.55), which were used to establish a nomogram model. The bootstrap-corrected C-index of the nomogram was 0.733 (95% CI = 0.68–0.79), which was significantly higher than that of each individual factor. The calibration curve and decision curve showed good consistency and clinical net benefit of the model. At the maximum Youden index, 49.4% (43/87) of women in the high-risk group defined by nomogram had concurrent EC, versus 16.6% in the low-risk group (*P*< 0.001).

**Conclusion:**

The nomogram based on HE4, menopausal status, and BMI was found with an improved predictive value to stratify preoperative-EAH patients at high risk of concurrent EC for better clinical management.

## 1 Introduction

Endometrial atypical hyperplasia (EAH) is the precursor lesion of endometrial cancer (EC) (1). For women diagnosed with EAH through endometrial biopsy (preoperative-EAH), hysterectomy and bilateral salpingectomy are the recommended first-line treatment if fertility preservation is not a consideration (2). However, 10%–63% of patients with preoperative-EAH were found to have concurrent EC after definitive hysterectomy (3–7), and the restaging surgery was required for high-risk patients to evaluate lymph node metastasis and then guide the adjuvant therapy (8). Unfortunately, due to the lymphatic channel disruption by the initial surgery, sentinel lymph node (SLN) assessment with fewer adverse events would not be an option. Under this circumstance, patients with concurrent EC could only undergo systematic lymphadenectomy which might cause lymphedema and long-term recovery time. However, one study that analyzed 268 preoperative-EAH patients who underwent unilateral or bilateral SLN assessment revealed 12 sentinel lymph node metastases (4.5%) including 2 macro-metastases, 9 micro-metastases, and 1 isolated tumor cells (9). This result indicated that despite providing more prognostic information, SLN assessment might not be suitable for all preoperative-EAH patients, due to the risk of overtreatment and higher cost. Thus, it is very important to more precisely identify preoperative-EAH patients with coexisting EC for better treatment plan, and to diminish the risks associated with the second anesthesia and systematic lymph node assessment.

Increased thickness of endometrium, postmenopausal status, elevated serum level of cancer antigen 125 (CA125), older age, suspicion of cancer on hysteroscopy (detailed criteria include endometrial growth showing a friable consistency with focal necrosis and atypical vessels, and endometrial growth could be papillary, polypoid, nodular, or mixed type), increased body mass index (BMI), diabetes comorbidity, and other factors were found to be correlated with concurrent EC in preoperative-EAH patients (5, 10–16). A retrospective cohort study reported that endometrial thickness ≥15 mm was associated with an increased risk of concurrent EC (10), whereas a large Danish retrospective study found that risk factors of EC among EAH women were age and menopause, especially the latter (13). In a previous retrospective study, under an expert pathological review, we found that women with postmenopausal status and elevated serum level of CA125 might have a high risk of concurrent EC, especially those with postmenopausal time ≥ 5 years (14). EAH has been stratified based on integrated histological parameters (17) to assess the risk of EC that was five times higher in the high-grade group than in the low-grade group (50% vs. 9.5%). However, there is no consensus on risk factors or predictive models, as previous studies lack central review of pathology on preoperative diagnosis, or these risk markers have not yet shown practical value in clinical use. In addition, serum biomarkers have been considered as potential predictive markers in clinical settings because they are easily accessible and less invasive. However, only a few serum biomarkers have been investigated in early studies without including human epididymis protein 4 (HE4), which have been shown to increase significantly in patients with EC (18–21).

This study aimed to investigate the risk factors including HE4 that might predict concurrent EC in preoperative-EAH patients from a tertiary hospital with pathology central review, and then to establish a nomogram prediction model to help stratify high-risk patients for better clinical management.

## 2 Materials and methods

### 2.1 Study population

The population for this retrospective cohort study consisted of 370 patients preoperatively diagnosed with EAH, who received definitive hysterectomy between January 2020 and December 2022 at the Obstetrics and Gynecology Hospital of Fudan University, Shanghai, China. The following are the inclusion criteria for patients to be eligible: (1) were preoperatively diagnosed with EAH by endometrial biopsy through Pipelle or dilation and curettage (D&C) with or without hysteroscopy (HSC)—all the endometrial biopsies were performed as operating room procedures; (2) underwent definitive hysterectomy within 3 months after endometrial biopsy; (3) had not received fertility-sparing treatment within 6 months before hysterectomy; (4) had no other malignant tumors; (5) had available clinicopathological data and information of serum HE4 within 1 month before definitive hysterectomy.

The study received approval from the Ethics Committees of the Obstetrics and Gynecology Hospital (protocol number 2021-185), and all the enrolled patients provided approval forms regarding collecting their medical information and laboratory data for research purposes.

### 2.2 Pathological diagnosis

The pathological diagnoses were determined by two senior gynecological pathologists at the hospital following the World Health Organization (WHO) pathological classification of tumors of the uterine corpus (2020) (22). If the diagnosis differed, a consultation would be conducted by pathologists to reach a final decision.

The diagnosis of EC after the definitive hysterectomy was restaged according to the latest version (2023) of the International Federation of Gynecology and Obstetrics (FIGO) staging system (23).

### 2.3 Data collection and evaluation

Clinical data were collected from the medical record including age, height, weight, menopausal status, fertility, comorbidities, details from the ultrasound evaluation, and endometrium biopsy (Pipelle or D&C with or without HSC). Patients’ metabolic status was evaluated by laboratory data, including fasting blood glucose (FBG) measured in venous plasma by using the glucose oxidation method within 4 h, and serum creatinine (Scr) measured by using a Hitachi 7600 automatic chemistry analyzer (Hitachi Diagnostics Ltd.). Additionally, the levels of serum tumor markers including HE4 and CA125 were measured by a Roche COBAS e 601 electrochemiluminescence analyzer (Roche Diagnostics Ltd.). A pathological report of endometrial biopsy and hysterectomy was also collected.

BMI was calculated as weight (kg)/height (m^2^). Moreover, the estimated glomerular filtration rate (eGFR) index was calculated using the following formula: eGFR (ml/min/1.73 m^2^) = 175 × [Scr (mg/dl)^−1.234^] × [age (years)^−0.179^] × 0.79 according to the Chinese eGFR Investigation Collaboration (24). BMI ≥ 28 kg/m^2^ was considered obese based on the Chinese population standard (25), whereas eGFR< 90 ml/min/1.73 m^2^ was identified as impaired renal function (26). The optimal cutoff value for HE4 was analyzed and selected using receiver operating characteristic (ROC) curves with a maximum value of Youden index (Youden index = sensitivity + specificity − 1).

### 2.4 Statistical analysis

Statistical analysis was performed by using SPSS 25.0 (SPSS, Chicago, USA) and R language (version 4.1.2) for Windows. The Shapiro–Wilk test or Kolmogorov–Smirnov test was utilized to determine whether continuous variables followed a normal distribution. Variance homogeneity was evaluated by Levene’s test. Normally distributed continuous variables were expressed as $\bar{x}$ ± s, or medians and interquartile range (IQR) when not normally distributed. The comparisons between final-EAH and final-EC groups of continuous variables were assessed by independent samples t test for normally distributed variables, or Mann–Whitney U tests for non-normally distributed variables. Categorical variables were presented as frequency with percentage, and comparisons between two groups were assessed by Chi-squared test or Fisher’s exact test depending on appropriate assumptions.

The logistic regression model was used for univariate and multivariate analyses to find independent predictive factors of concurrent EC. Variables with *P*< 0.05 in univariate analysis or with clinical significance were included into multivariate analysis in which the forward likelihood ratio method was applied. Adjusted odds ratio (OR) and 95% confidence intervals (CIs) were estimated with the logistic regression models.

Based on the multivariate logistic regression model, a nomogram was visually established. The goodness of fit was evaluated by using the Akaike information criterion (AIC) and Bayesian information criterion (BIC). The prediction accuracy was evaluated by using the bootstrap-corrected concordance index (C-index). The consistency and clinical net benefit were evaluated by the calibration curve and decision curve analysis (DCA), compared with factors for predicting concurrent EC which enrolled into multivariate analysis. All internal verifications were conducted by using bootstrapping method with 1,000 resamples. The nomogram, calibration curves, and DCA curves were plotted by the *rms* package in R.

According to the nomogram, each preoperative-EAH patient obtained an individual risk score. An optimal cutoff point for the risk score which provided the maximum Youden index for prediction of final-EC was calculated. It measured the effectiveness and provided sufficient confidence that the nomogram could be used in a clinical setting to identify high-risk patients with coexisting EC. Then, the optimal cutoff was used to stratify these patients into low-risk and high-risk groups for concurrent EC.

A two-tailed *P* value of less than 0.05 was considered statistically significant.

## 3 Results

**Figure 1:**
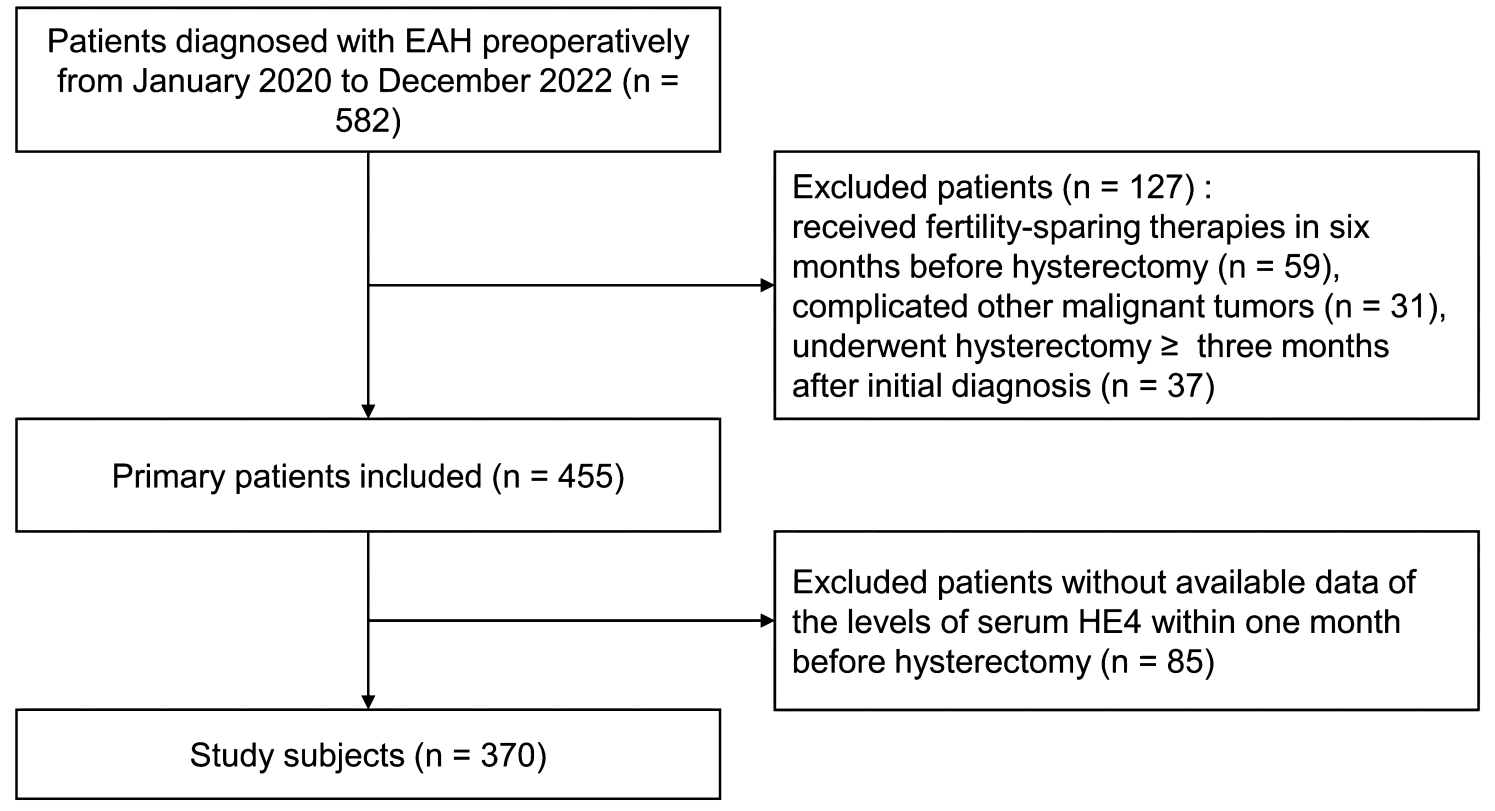
The flowchart of patient selection. EAH, endometrial atypical hyperplasia; HE4, human epididymis protein 4.

We retrospectively screened 582 preoperative-EAH patients who were diagnosed with EAH or EC based on conclusive histopathological examinations after hysterectomy (final-EAH or final-EC). Eventually, 370 of them met the study criteria and were included in the analysis (**Figure 1**). Approximately one quarter (24.3%, 90/370) of the cohort had concurrent EC, which were all endometrioid histology subtype. Patients’ clinicopathological characteristics are shown in **Table 1**.

**Table 1:** Clinico-pathological characteristics for patients diagnosed with EAH and EC by final histopathology (n = 370).

Characteristics	Number	Total patients (n = 370)	Final-EAH (n = 280)	Final-EC (n = 90)	*P* value
Age (years)	370				
Median (IQR)		47 (41-51)	46 (41-51)	48 (44-55)	0.004
≤40 (n, %)		86 (23.2)	69 (24.6)	17 (18.9)	0.001
41-50 (n, %)		177 (47.8)	140 (50.0)	37 (41.1)	
51-60 (n, %)		89 (24.1)	64 (22.9)	25 (27.8)	
>60 (n, %)		18 (4.9)	7 (2.5)	11 (12.2)	
BMI (kg/m^2^)	370				
Median (IQR)		24.56 (22.43-27.56)	24.46 (22.41-27.14)	24.90 (22.47-30.29)	0.161
<28 (n, %)		284 (76.8)	224 (80.0)	60 (66.7)	0.009
≥28 (n, %)		86 (23.2)	56 (20.0)	30 (33.3)	
Menopausal status (n, %)	370				
Premenopausal		300 (81.1)	244 (87.1)	56 (62.2)	<0.001
Postmenopausal time< 5 years		40 (10.8)	25 (8.9)	15 (16.7)	
Postmenopausal time ≥ 5 years		30 (8.1)	11 (3.9)	19 (21.1)	
Fertility (n, %)	370				
Pluripara		346 (93.5)	263 (93.9)	83 (92.2)	0.567
Nullipara		24 (6.5)	17 (6.1)	7 (7.8)	
Diabetes (n, %)	360				
NO		321 (89.2)	248 (90.5)	73 (84.9)	0.143
YES		39 (10.8)	26 (9.5)	13 (15.1)	
Hypertension (n, %)	314				
NO		229 (72.9)	176 (74.9)	53 (67.1)	0.177
YES		85 (27.1)	59 (25.1)	26 (32.9)	
FBG (mmol/L) (n, %)	357				
<7.0		336 (94.1)	253 (94.4)	83 (93.3)	0.691
≥7.0		21 (5.9)	15 (5.6)	6 (6.7)	
HE4 (pmol/L)	370				
Median (IQR)		45.79 (39.30-52.73)	43.70 (38.25-50.98)	50.50 (44.28-60.73)	<0.001
CA125 (U/ml)	361				
Median (IQR)		15.30 (11.77-21.64)	15.50 (11.88-22.19)	14.87 (11.50-20.75)	0.738
<35 (n, %)		321 (88.9)	243 (88.7)	78 (89.7)	0.802
≥35 (n, %)		40 (11.1)	31 (11.3)	9 (10.3)	
eGFR (ml/min/1.73 m^2^)	366				
Median (IQR)		133.13 (116.74-150.64)	134.77 (118.45-150.73)	129.05 (112.96-150.82)	0.339
≥90 (n, %)		357 (97.5)	270 (97.8)	87 (96.7)	0.822
<90 (n, %)		9 (2.5)	6 (2.2)	3 (3.3)	
Sampling method (n, %)	370				
D&C alone		166 (44.9)	128 (45.7)	38 (42.2)	0.761
D&C with HSC		191 (51.6)	143 (51.1)	48 (53.3)	
Pipelle biopsy		13 (3.5)	9 (3.2)	4 (4.4)	
Final-EC patients (n=90)					
Histology (n, %)	90				
Endometrioid cancer		/	/	90 (100)	/
Other histological type				0(0)	
Re-stage by FIGO version 2023 (n, %)	90				
I		/	/	84 (93.3)	/
II		/	/	4 (4.4)	
III		/	/	2 (2.2)	
Grade (n, %)	88				
G1		/	/	85 (96.6)	/
G2		/	/	2 (2.3)	
G3		/	/	1 (1.1)	
Myometrial invasion (n, %)	90				
No or<50%		/	/	81 (90.0)	/
≥50%		/	/	9 (10.0)	
LVSI (n, %)	90				
(−)		/	/	82 (91.1)	/
(+)		/	/	8 (8.9)	
Sentinel lymph node (n, %)	15				
(−)		/	/	14 (93.3)	/
(+)		/	/	1 (6.7)	
MELF (n, %)	90				
(−)		/	/	85 (94.4)	/
(+)		/	/	5 (5.6)	

Data were presented as number (%) or median (interquartile range). Percentage calculations excluded missing data. Missing data included 10 cases for diabetes, 56 for hypertension, 13 for FBG, 9 for CA125, and 4 for eGFR value. P value: difference between the final-EAH group and final-EC group. Significant P value< 0.05.

EAH, endometrial atypical hyperplasia; EC, endometrial cancer; final-EAH, endometrial atypical hyperplasia diagnosed by final histopathology; final-EC, endometrial cancer diagnosed by final histopathology; IQR, interquartile range; BMI, body mass index; FBG, fasting blood glucose; HE4, human epididymis protein 4; CA125, cancer antigen 125; eGFR, estimated glomerular filtration rate; D&C, dilatation and curettage; HSC, hysteroscopy; LVSI, lymphovascular space invasion; FIGO, the International Federation of Gynecology and Obstetrics; MELF, microcystic, elongated, and fragmented.

Compared with the final-EAH group, more patients in the final-EC group were older (*P* = 0.004), obese (*P* = 0.009), and in postmenopausal status (*P<* 0.001). Notably, the median level of serum HE4 significantly increased in the final-EC group than in the final-EAH group (50.5 pmol/L vs. 43.7 pmol/L, *P*< 0.001, **Table 1**). Nevertheless, no significant intergroup difference was observed in other factors such as CA125, eGFR, fertility history, diabetes, hypertension, FBG, and endometrial sampling methods.

In the ROC curve, the serum HE4 level also showed a substantial predictive potential for detecting coexisting EC (AUC = 0.689, 95% CI = 0.63–0.75, *P*< 0.001, in **Supplementary Figure 1**). The optimal cutoff value was determined at 43.50 pmol/L based on the maximum Youden index at 0.296 with sensitivity of 0.800 and specificity of 0.496 (**Supplementary Figure 1**).

**Figure 2:**
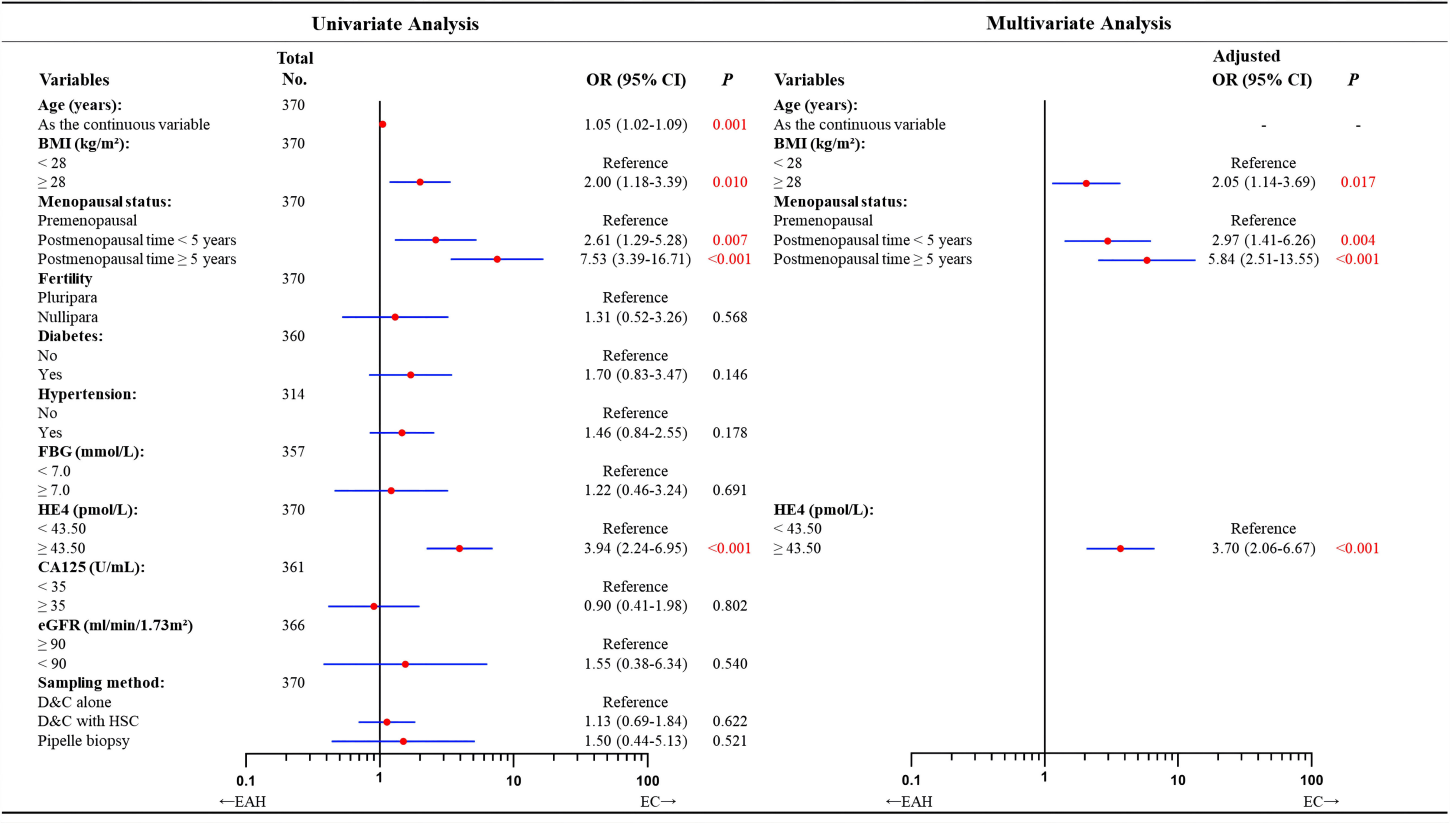
Univariate and multivariate analyses of factors predicting concurrent EC in patients diagnosed with EAH preoperatively according to logistic regression model. In total of 370 patients with available data. Missing data included 10 cases for diabetes, 56 for hypertension, 13 for FBG, 9 for CA125, and 4 for eGFR. According to the forward likelihood ratio regression results, the multivariate logistic model containing BMI, menopausal status, and HE4 value in the preoperative-EAH patients. Although age was found to correlate with final-EC in the univariate analysis, it was excluded in the multivariate modeling with forward likelihood ratio selection. OR adjusted for BMI, menopausal status, and HE4 value. Significant *P* value< 0.05. EAH, endometrial atypical hyperplasia; EC, endometrial cancer; BMI, body mass index; FBG, fasting blood glucose; HE4, human epididymis protein 4; CA125, cancer antigen 125; eGFR, estimated glomerular filtration rate; D&C, dilatation and curettage; HSC, hysteroscopy; OR, odds ratio; CI, confidence interval.

In univariate analysis, we found the predictive factors for coexisting EC were older age (OR = 1.05; 95% CI = 1.02–1.09; *P* = 0.001), obesity (OR = 2.00; 95% CI = 1.18–3.39; *P* = 0.010), higher level of serum HE4 (OR = 3.94; 95% CI = 2.24–6.95; *P*< 0.001), and longer postmenopausal time (postmenopausal time< 5 years vs. premenopausal status: OR = 2.61, 95% CI = 1.29–5.28, *P* = 0.007; postmenopausal time ≥ 5 years vs. premenopausal status: OR = 7.53, 95% CI = 3.39–16.71, *P*< 0.001). However, the independent predictors remained by multivariate analysis as: HE4 ≥ 43.50 pmol/L (OR = 3.70; 95% CI = 2.06–6.67; *P*< 0.001), BMI ≥ 28 kg/m^2^ (OR = 2.05; 95% CI = 1.14–3.69; *P* = 0.017), postmenopausal time< 5 years (OR = 2.97; 95% CI = 1.41–6.26; *P* = 0.004) and postmenopausal time ≥ 5 years (OR = 5.84, 95% CI = 2.51–13.55; *P*< 0.001, **Figure 2**).

**Figure 3:**
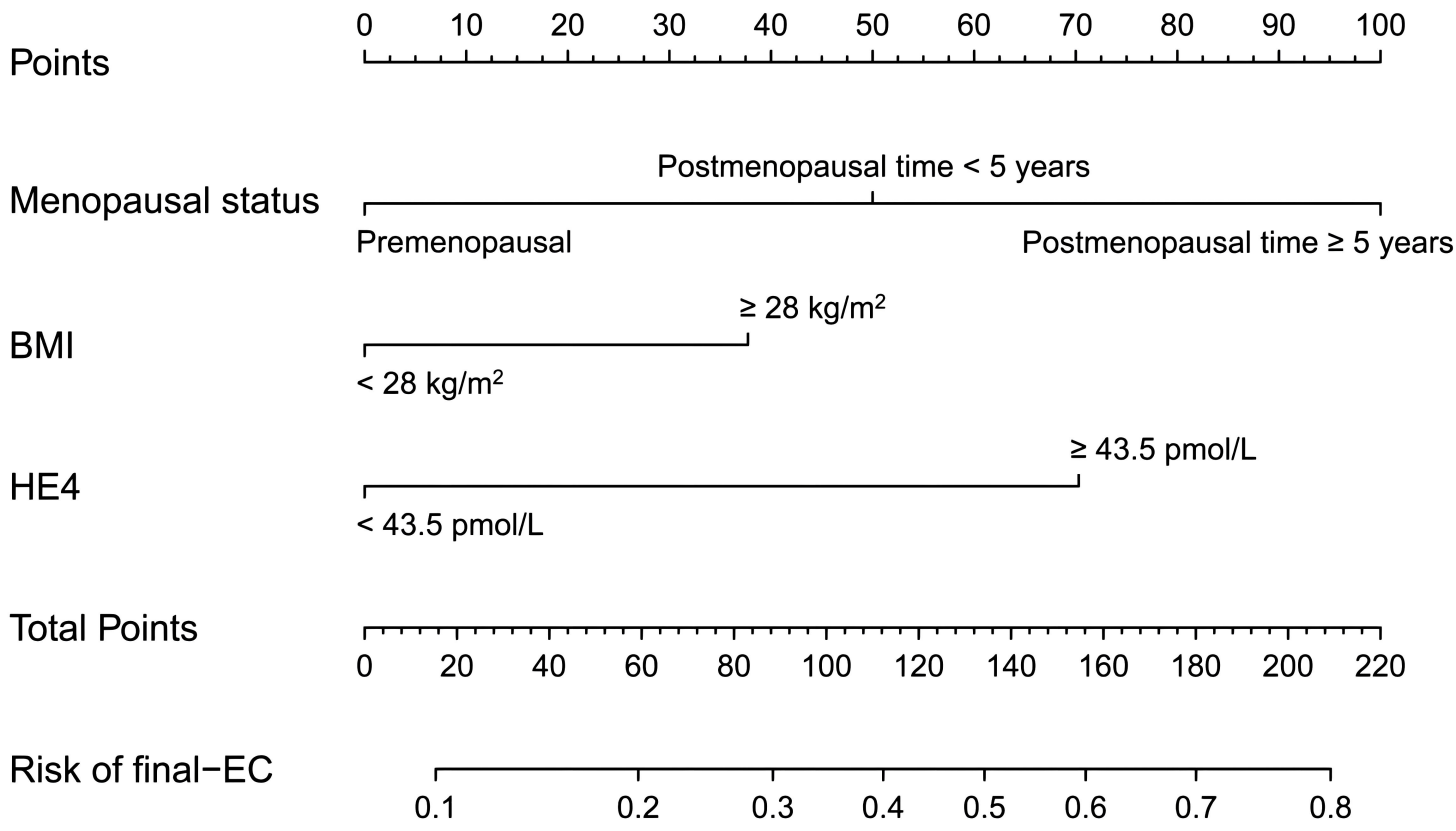
The developed nomogram based on menopausal status, BMI, and HE4 for predicting concurrent EC in preoperative-EAH patients. EC, endometrial cancer; EAH, endometrial atypical hyperplasia; HE4, human epididymis protein 4; BMI, body mass index; C-index, concordance index; CI, confidence interval; AIC, Akaike Information Criterion; BIC, Bayesian Information Criterion.

**Figure 4:**
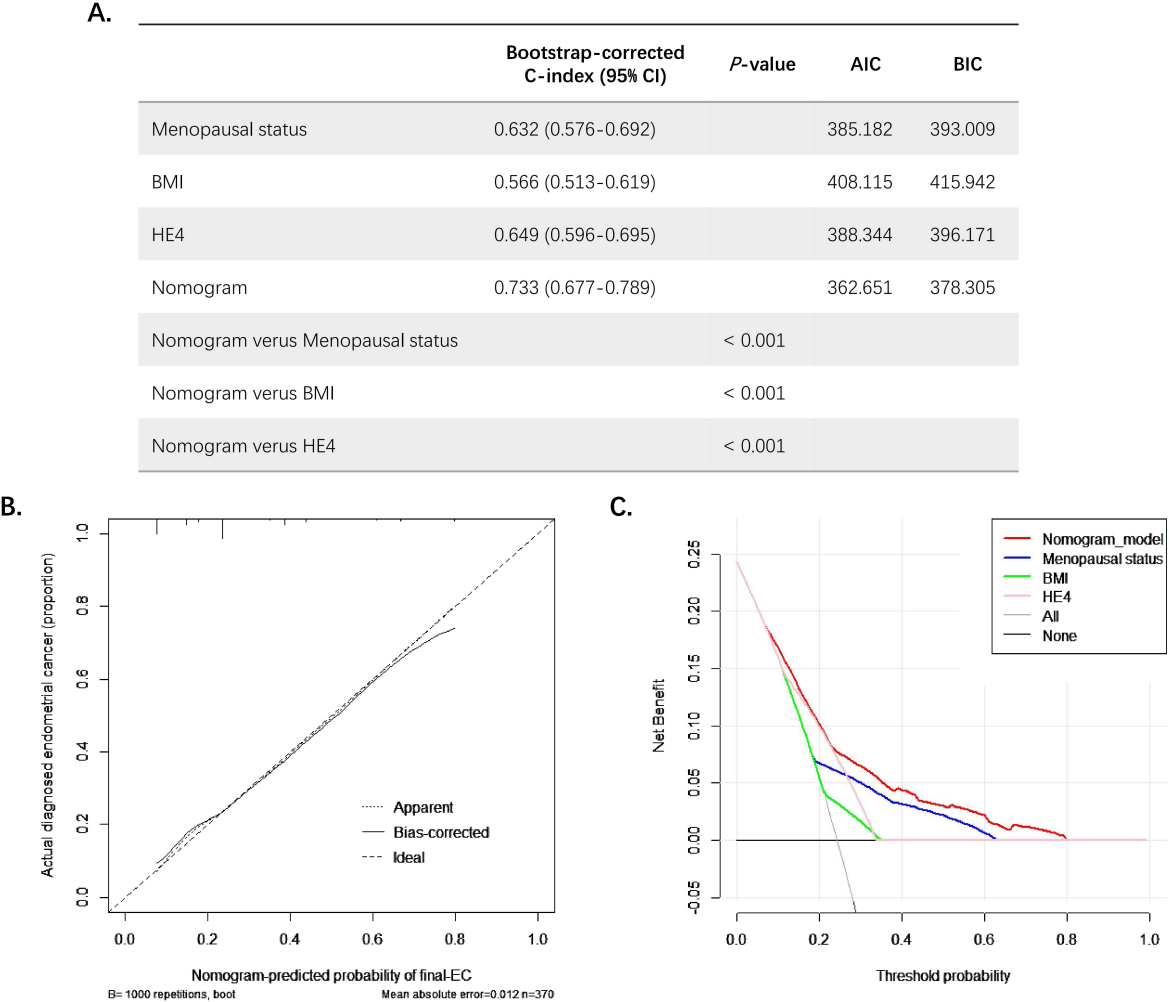
The examination of goodness-of-fit ability, discriminative ability, net benefit, and predictive capacity of the nomogram. **(A)** The AIC, BIC, and bootstrap-corrected C-index of prognostic factors and nomogram for predicting concurrent EC. **(B)** The calibration curves of the nomogram prediction. **(C)** The decision curve analysis of nomogram compared with menopausal status, BMI, and HE4 for predicting concurrent EC. Significant *P* value< 0.05. EC, endometrial cancer; EAH, endometrial atypical hyperplasia; HE4, human epididymis protein 4; BMI, body mass index; C-index, concordance index; CI, confidence interval; AIC, Akaike Information Criterion; BIC, Bayesian Information Criterion.

Then, a nomogram was subsequently developed based on the three independent factors (**Figure 3**). Remarkably, in terms of distinguishing evaluation, the C-index of the nomogram was 0.733 for the primary cohort and remained 0.733 (95% CI = 0.68–0.79) through bootstrap-corrected validation, which was higher than that of each individual factor alone (**Figure 4A**). Meanwhile, a goodness of fit of the nomogram was also found because the model’s AIC and BIC (362.65 and 378.31, respectively) were both lower than that of each predictive marker alone. For the consistency evaluation, the calibration curve revealed a good agreement between prediction and observation for the probability of final EC among the 370 preoperative-EAH patients (**Figure 4B**). Furthermore, the DCA showed if the threshold probability of a patient is between 8% and 79%; the utilization of the nomogram to predict the final-EC in preoperative-EAH patients yielded a higher net benefit, outperforming both the all-patients scheme and the none scheme (**Figure 4C**).

**Figure 5:**
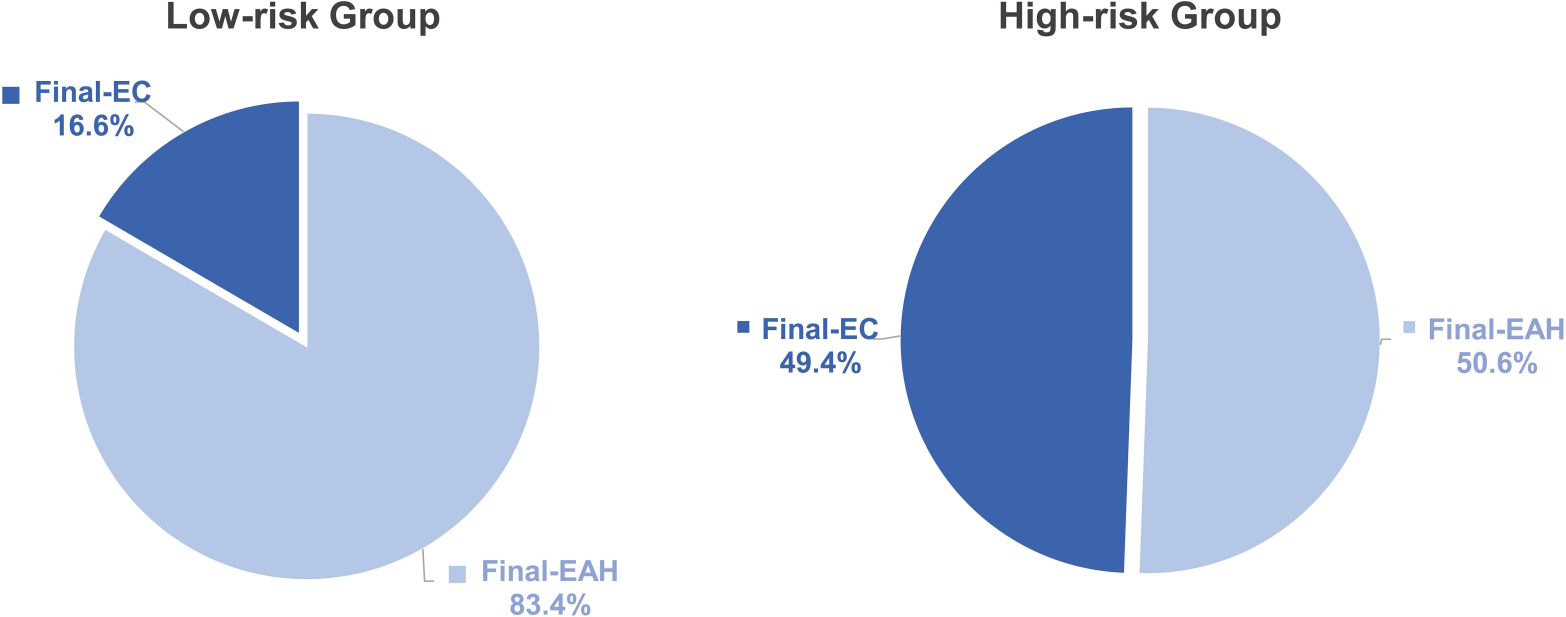
The distribution of final-EC in low- and high-risk groups based on the points of nomogram. Low-risk, total points<93.9; high-risk, total points ≥ 93.9. P value: difference between low-risk group and high-risk group. Significant *P* value< 0.05. EC, endometrial cancer; EAH, endometrial atypical hyperplasia.

Calculating each patient’s total risk point from nomogram, the cutoff value was determined at 93.9 points providing a maximum Youden index and was used to stratify all 370 preoperative-EAH patients into low-risk and high-risk subgroups to compare the risk differences of concurrent EC. It showed that 49.4% (43/87) of patients in the high-risk group were finally diagnosed with EC, whereas less than one-fifth (16.6%, 47/283) of low-risk patients had final EC (*P<* 0.001, **Figure 5**). The sensitivity, specificity, positive predictive value, and negative predictive value were 0.478, 0.843, 0.494, and 0.834, respectively.

## 4 Discussion

In this study, serum level of HE4 ≥ 43.50 pmol/L, BMI ≥ 28 kg/m^2^, and postmenopausal status were found as independent predictors for concurrent EC. An easy-to-use nomogram model was constructed by incorporating these factors, which significantly improved the predictive value of each individual factor alone. Meanwhile, the model was internally validated with a good consistency and clinical net benefit. At the maximum Youden index, the model significantly stratified preoperative-EAH patients with high or low risks of concurrent EC (49.4% vs. 16.6%).

Our findings have important clinical implications, as currently there is no predictive model that has been developed as a tool to aid clinical decision-making for preoperative-EAH women (5, 10–13). In most situations, preoperative-EAH patients were suggested to take definitive surgery without further evaluation, such as pelvic magnetic resonance imaging (MRI) or computerized tomography (CT), due to the high cost and radiological side effect. In our study, 90 of 370 (24.3%) preoperative-EAH patients had concurrent EC. It meant that 370 patients all underwent initial hysterectomy + salpingectomy and then 90 final-EC patients were incompletely staged. When second surgery required, more injury and complications were generated. Conversely, if these 370 patients all underwent staging surgery considering the estimated risk of final-EC, then 280 final-EAH patients would be overtreated. Therefore, using our nomogram, we might provide the high-risk patients with a more appropriate treatment plan, including presurgical evaluation of MRI or CT and suggesting hysterectomy + salpingectomy + oophorectomy + sentinel lymph node assessment as initial surgery, considering almost half of these patients (49.4%) might have concurrent EC. On the other side, since the risk of concurrent EC was nearly one out of six (16.7%) for low-risk patients, oophorectomy and staging surgery might not be suggested to avoid bringing more loss than benefit, particularly for premenopausal women. Hence, with nomogram, we could provide more individualized treatment to prevent from overtreatment or undertreatment. Also, with the simple chart of nomogram, it is easy to calculate the individual score of each patient to assess the potential risk of cancer. Notably, almost a half (49.4%) of high-risk patients by this nomogram had concurrent EC, showing the model’s usefulness in clinical stratification. The bootstrap-corrected C-index of 0.733 (95% CI = 0.68–0.79) indicated a good discrimination which was better than chance (AUC > 0.7). Nevertheless, the nomogram should not displace the clinical judgment of surgeons, but it could be utilized as a good complementary tool for treatment plan development or during the consultation with the patients. Additionally, the nomogram can be used to stratify or randomize patients according to their risk levels in future clinical trials focused on concurrent endometrial cancer in preoperative atypical endometrial hyperplasia.

We reported that 24.3% (90/370) of preoperative-EAH patients had concurrent EC that was lower than previously presented (3, 6, 10, 11, 13), probably due to the tertiary-hospital-based pathological review on the initial and final diagnoses of EAH and EC, which improved the accuracy of the patient selection. Distinguishing EAH from well-differentiated EC remains difficult in pathology. Firstly, the use of different histologic criteria can lead to divergent pathological assessments, including WHO 2014 or Endometrial Intraepithelial Neoplasia criteria coupled with varying diagnostic thresholds (27). Secondly, it is difficult to differentiate the histologic features indicative of muscular invasion, which are crucial for diagnosing EC (28, 29). Additionally, technical issues and subjectivity can influence diagnostic accuracy, including inadequate clinical data, insufficient sampling, inappropriate fixation, and poor staining quality (1, 27). Pathologists in our tertiary hospital of obstetrics and gynecology are specialized in gynecology and obstetrics, including gynecological oncology, with long-term experience in evaluating endometrial tissues. In our study, all the pathological diagnoses were determined by at least two senior gynecological pathologists in our hospital. If the diagnoses were inconsistent, a consultation would be held in the department of pathology to reach a final decision, to minimize the diagnostic bias on assessing risk factors for coexisting EC.

Menopausal status was one of the predictors of concurrent EC in our study, which was consistent with previous findings (5, 10–13). A large-scale (n = 773) Danish study supported the similar predictive value of menopausal status based on a community-based clinical database without pathological review on the pre- and postsurgical diagnoses (13). This was further confirmed by our results after the central review by gynecological pathologists at a tertiary hospital. We additionally reported that the risk of concurrent EC increased in patients with long postmenopausal time (especially ≥ 5 years), which was also identified by our previous study (14).

Our analysis did not include endometrial thickness evaluated by preoperative ultrasound. Recently, Abt et al. demonstrated that increased endometrial thickness might be associated with a higher risk of coexisting EC among 378 patients with endometrial intraepithelial neoplasia (10). The median age of that study was 55 (range 48–62) years, whereas the menopausal status of these patients was not mentioned or adjusted (10). Another retrospective study (n = 70) indicated that endometrial thickness measured by transvaginal ultrasonography was not statistically different between patients with unexpected EC and those with EAH postoperatively (30). Rajadurai et al. also found that endometrial thickness was irrelevant to the risk of concurrent EC in patients with an initial diagnosis of EAH before surgery (n = 97) (11). In this regard, transvaginal ultrasound seemed to be unreliable to detect concurrent EC before surgery. Considering that our research included both pre- and postmenopausal women, and the potential bias of endometrial thickness evaluated by various clinicians in different time points of menstrual cycles, we did not incorporate this factor into our model.

To our knowledge, serum HE4 was for the first time incorporated into the nomogram model to predict concurrent EC for preoperative-EAH women in our study. HE4 was a glycoprotein mainly expressed in the epididymis and found in other tissues such as the respiratory and reproductive tracts (31, 32). Increasing findings showed a significant correlation between the HE4 and EC (18–21, 33). Knific et al. found that the serum HE4 level was significantly higher in EC patients compared with patients with benign gynecological diseases, indicating its diagnostic potential (18). A meta-analysis supported serum HE4 as a potential biomarker for EC with a pooled sensitivity and specificity of 0.65 and 0.90, respectively (21). So far, the most commonly studied biomarker for clinical management of EC was CA125 (18, 34). An elevated serum level of CA125 was found to be correlated with concurrent EC in preoperative-EAH patients (14). However, several researchers demonstrated that serum CA125 might be a unreliable biomarker for detecting EC (18, 35, 36), due to its poor sensitivity and specificity in distinguishing EC from normal endometrium (35) and the inability to differentiate EC and non-malignant endometrial pathologies (36). It is reported that HE4 has been considered to be a more accurate and sensitive serum biomarker than CA125 to distinguish early EC and normal endometria (20). Notably, it was found that HE4 increased in all stages of EC and had better diagnostic sensitivity for early-stage EC compared with CA125, whereas CA125 elevated in late stages of EC as well as in women with endometriosis and pelvic infectious diseases (19, 37–39). In our multivariate analysis, serum HE4 rather than CA125 was relevant to concurrent EC, which provided further evidence of the better predictive value of HE4 in this population.

From our analysis, serum HE4 at a cutoff of 43.50 pmol/L revealed its predictive value of coexisting EC and EAH and then contributed to the nomogram model for better clinical use. Currently, the cutoff of the serum HE4 level has been mainly investigated in ovarian cancers, but no consensus has been reached for endometrial cancer. It should be noted that the cutoff of HE4 might fluctuate in different malignancies, and further verifications in prospective studies are needed.

BMI ≥ 28 kg/m^2^ was found as a significant predictor for final-EC diagnosis among preoperative-EAH patients in this study. It is well recognized that BMI is strongly associated with EC and EAH (2). Erdem et al. found higher BMI as a potential risk factor for EC presence in preoperative-EAH patients (n = 227), but when BMI was transformed into a categorical variable with a set at 30 kg/m^2^, the predictive value was lost (40). On the other hand, another study in Turkey reported that BMI ≥ 30 kg/m^2^ was an independent predictor for coexisting EC in patients with complex EAH preoperatively (n = 128) (16). However, other research did not support the significance of BMI (12, 13). Antonsen et al. divided BMI into groups of<18.5 kg/m^2^, 18.5 kg/m^2^–25.0 kg/m^2^, 25.0 kg/m^2^–35.0 kg/m^2^, and ≥35.0 kg/m^2^, but no significant value was found to predict concurrent EC in preoperative-EAH women in Denmark (n = 773) (13). This inconsistency among studies might be explained by the differences in sample sizes, population characteristics, and the cutoff of BMI.

Previous studies have explored the factors associated with concurrent EC in patients with preoperative EAH. However, no consensus has been reached on risk factors, and prediction models with relevant validation were lacking. Giannella L et al. employed regressions and artificial neural networks to retrospectively predict EC in 629 preoperative-EAH patients (41). Only patients’ characteristics were analyzed in that study showing no significant predictive value. The authors suggested that patients’ characteristics alone were inadequate for predictive model, and more serum chemical markers should be further investigated (41). In our nomogram, we incorporated patients’ characteristics along with serum biomarkers evaluated by laboratory data, which presented excellent consistency and clinical net benefit with a bootstrap-corrected C-index of 0.733, suggesting its good predictive value of EC and easy use in a clinical setting. A Brazilian study also developed a nomogram to predict the risk of endometrial cancer and precursor lesion for patients on the hysteroscopy waiting list to optimize the prioritization of hysteroscopy procedures (42). The nomogram includes factors such as age, BMI, postmenopausal bleeding, pregnancy history, hypertension, diabetes, and uterine volume endometrial thickness showing a good stratification that women who had nomogram score >197 might have >90% risk of endometrial cancer or precursor lesion. However, this study focused on predicting patients who may have endometrial abnormalities after hysteroscopic evaluation, which was different to our nomogram aiming to predict the risk of concurrent EC after definitive surgery for EAH patients who already diagnosed by endometrial biopsy. Our study notably established an easily used nomogram to improve the clinical treatment strategy for preoperative EAH patients to achieve better prognosis and less surgical complications.

In our nomogram, we only included the clinicopathological characteristics and serum biomarkers. However, recent research using single-cell RNA sequencing and revealed that seven signature genes were significantly upregulated in both EAH and EEC, but three genes of them (DKK4, CST1, and NOTUM) were only highly expressed in EEC rather than in EAH (43). These findings indicated that DKK4, CST1, and NOTUM might be with the great value as biomarkers to detect concurrent EC from EAH. These genes might be considered in the future study to enhance the predictive power of the nomogram in this field.

Our study has several limitations. Although with a relatively large sample size, the results are from a retrospective single-center study. Only internal verification has been performed without subsequent external validation, which might impact the generalizability of our findings. Moreover, the model was established based on three predictive factors with a bootstrap-corrected C-index at 0.733, leaving room for improvement with a better discrimination in future studies. In addition, we did not analyze high-intermediate-risk EC due to limited number of patients. To resolve these limitations, further external verification and prospective multicenter studies are needed in the future to include high-intermediate-risk EC. More potential biomarkers should be explored, to improve the predictive accuracy and clinical feasibility of the model.

In conclusion, this study established a nomogram based on HE4, menopausal status, and BMI with improved predictive value and clinical convenience. The nomogram might help to stratify preoperative-EAH women with high risk of concurrent EC for better clinical decision-making and individualized management. Nevertheless, the findings should be further validated in prospective multicenter studies. Also, more less-invasive biomarkers should be investigated for this population and included into predictive models.

## Data Availability

The raw data supporting the conclusions of this article will be made available by the authors, without undue reservation.
