# Supplementary figures and images for: Nomogram using human epididymis protein 4 predicted concurrent endometrial cancer from endometrial atypical hyperplasia before surgery
Source: Front Oncol. 2024 Sep 6;14:1442127. doi: 10.3389/fonc.2024.1442127 (PMC11412798; doi:10.3389/fonc.2024.1442127)

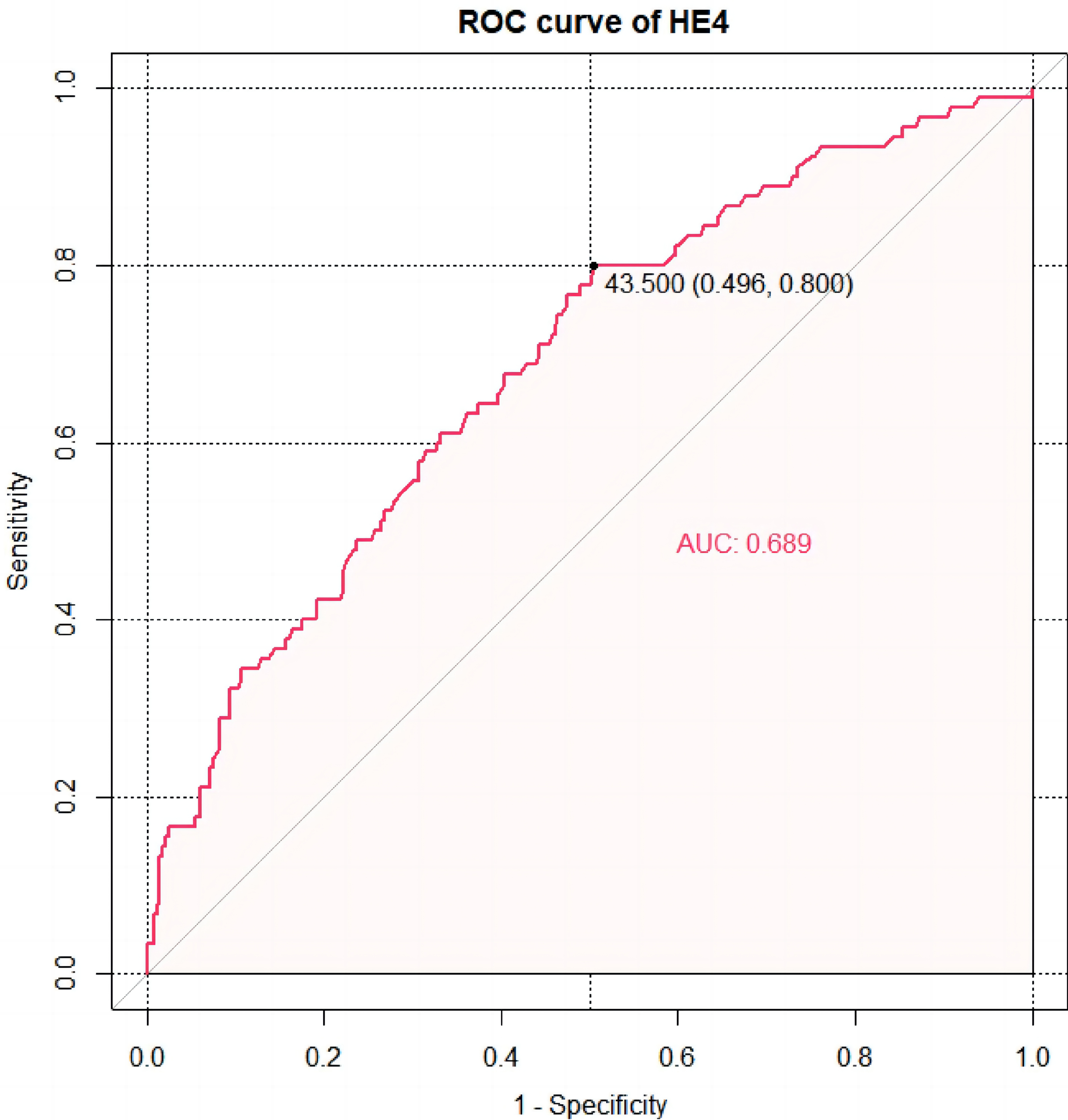

| Cutoff of serum-HE4 value | Specificity | Sensitivity | Youden index |
|---------------------------|-------------|-------------|--------------|
| 43.50                     | 0.496       | 0.800       | 0.296        |
| 45.65                     | 0.557       | 0.700       | 0.257        |
| 49.40                     | 0.700       | 0.556       | 0.256        |
| 57.15                     | 0.893       | 0.344       | 0.237        |

Supplement: Supplementary file 1 [file DataSheet1.pdf]
