# Supplementary material for: Nomogram using human epididymis protein 4 predicted concurrent endometrial cancer from endometrial atypical hyperplasia before surgery
Source: Front Oncol. 2024 Sep 6;14:1442127. doi: 10.3389/fonc.2024.1442127 (PMC11412798; doi:10.3389/fonc.2024.1442127)
Supplement: Supplementary file 2 [file DataSheet2.docx]

Supplementary Material

# Supplementary Material

**Supplementary Figure 1.** The ROC curve of serum HE4 levels predicting concurrent EC among all 370 preoperative-EAH patients with specificity and sensitivity of serum-HE4 at different cutoffs.

Notes: Youden index = sensitivity + specificity - 1.

Abbreviations: EAH, endometrial atypical hyperplasia; EC, endometrial cancer; HE4, human epididymis protein 4; ROC, receiver operating characteristic.
